# Knowledge and Attitudes Regarding Family Planning Options in Armenia

**DOI:** 10.1089/whr.2024.0005

**Published:** 2024-04-26

**Authors:** Lara Rostomian, Christopher John Kliethermes, Anke Hemmerling

**Affiliations:** ^1^ Department of Integrative Biology, University of California, Berkeley, Berkeley, California, USA.; ^2^ Obstetrics and Gynecology, Wayne State University School of Medicine, Detroit, Michigan, USA.; ^3^ University of California, Berkeley School of Public Health, Berkeley, California, USA.; ^4^ UCSF Bixby Center for Global Reproductive Health, San Francisco, California, USA.

**Keywords:** reproductive health, family planning, contraception, Armenia, global health, sexual education

## Abstract

**Objective:**

In many Transcaucasian and Middle Eastern populations, research in women's sexual and reproductive health remains limited, especially in Armenia despite recent political and cultural changes. This study explores the current state of family planning in Armenia while both highlighting the recent progress and identifying current barriers to reproductive health.

**Study Design:**

We conducted a mixed-methods study using both a quantitative survey and qualitative interviews with women and key informants in the field of women's sexual and reproductive health.

**Results:**

Armenian women are familiar with many types of contraception. The use of modern methods has increased but remains low. Sexual education for women is uncommon and often sought through independent online searches or books. We found no significant access barriers, however, a prevailing distrust in hormonal contraceptive methods left many women to rely on condoms and withdrawal. Although the majority of surveyed women (72%) believed having access to safe abortions was an important right, only 42% would consider having an abortion in the case of an unintended pregnancy. Interviewees highlighted the lack of sexual education, discrepancies in sexual and reproductive services between rural provinces and the urban capital city of Yerevan, as well as the need for information and the government's responsibility in this field.

**Conclusions:**

The lack of comprehensive sexual education in Armenia fuels misinformation regarding family planning options. One option we recommend is a government-funded sexual education program which begins as culturally sensitive, sex-positive education in schools and continues with counseling and support for women within the health care system.

## Introduction

The Republic of Armenia is situated in the mountainous Caucasus region between Asia and Europe, and, like many other Post-Soviet countries, has a complex history and relationship with family planning services. Many researchers have identified Armenia to fall in line with patterns seen in most Post-Soviet countries: skepticism surrounding hormonal contraception which results in low use of modern contraceptives and a high reliance on abortion to regulate reproduction.^1–4^ The most recent study addressing the family planning context in Armenia is decades old. At the time, the primary method of contraception was withdrawal, and the leading reason for the nonuse of modern contraception was a perceived low risk of pregnancy.^5^ Despite low use of modern contraception, over the last 20 years, the country's total fertility rate has been at 1.7 children per woman.^6^

In 2000, only 39% of Armenian women used contraceptive methods, and 14.4% used modern methods.The latest available demographic household survey from 2015 shows little change (36.7% of women on contraception, and 18.1% using modern methods).^6^ Compared to countries in similar stages of development, Armenia continues to have a low contraceptive prevalence rate but already transitioned to a low total fertility rate, which suggests a continued reliance on abortion in the country.^7–12^ However, by 2007, the abortion rate in Armenia reportedly had decreased to 1.8 per woman during her lifetime, a contradiction that warrants further investigation to understand unique situation in Armenia.^13^ Nevertheless, it is imperative to note that these data are more than a decade old, and it is plausible that there has been subsequent reduction in abortion rates in Armenia post-2007, which may be attributable to an enhanced adoption of alternative family planning methods.

The lack of recent literature leaves policy makers lacking evidence for informed decisions, and Armenian women more vulnerable to access barriers and to the consequences of unmet family planning needs.

This study sought to identify the current state of reproductive health and family planning in Armenia, while highlighting both the progress made in the recent years and identifying remaining barriers.^14^ We explored Armenian women's knowledge and attitudes regarding family planning options, as well as the reasoning forming their opinions. This study provides insight to policy makers and health care providers, and many of the findings may be applicable to other post-Soviet countries.

## Materials and Methods

Using both qualitative and quantitative methods, this study used three avenues of data collection: anonymous surveys of Armenian women of reproductive age, one-on-one interviews with Armenian women of reproductive age, and one-on-one interviews with key stakeholders in the field of reproductive health. The study received ethics approval from the Office for Protection of Human Subjects (OPHS) at the University of California at Berkeley (Protocol ID: 2019-03-11898).

### Recruitment and inclusion criteria

The recruitment followed a descriptive phenomenological approach with purposive and snowball sampling methods. With the support of Armenian family planning providers and advocates, recruitment of both women and key informants in Yerevan was conducted through the Women's Resource Center Armenia (WRCA), a nongovernmental women's rights organization with partnering centers in a number of locations. Recruitment outside of Yerevan was done in collaboration with WRCA's sister centers in the specified provinces. Women were eligible to participate in the survey and interviews if they were older than the age of 18 years. Male and female key informants were recruited among stakeholders working in the health care field.

### Data collection and analysis methods

Questions for the qualitative interview guide and survey were developed by the principal investigator (PI), L.R. (a native Armenian speaker) based on the research team's subject matter knowledge and findings from existing literature (Supplemental Files 1 and 2). The questions in the survey and interview guide were tested to ensure clarity and comprehensibility by five other native Armenian speakers recruited from a pool of native and diasporan Armenian students at the University of California, Berkeley. Interviews lasted between 30 minutes and an hour and were conducted in Armenian by L.R. (PI). Interviews were audiorecorded, transcribed, and translated to English. We conducted thematic content analysis following a deductive coding approach using DeDoose Software (DeDoose Version 8.3.17). The survey and interview questions focused on assessing women's knowledge and attitudes regarding family planning as well as general sexual and reproductive health.

The six domains explored included demographics, contraception (profile of use, reasons for nonuse, preferences, opinions and knowledge), sexual education, abortion, access to reproductive health, and reproductive health needs. After completing an informed consent process, the survey was made available to participants either online for completion at a place and time of their choice, or as a paper copy to be completed either at WRCA or at affiliated nongovernmental organization (NGO) centers. Significantly more survey participants were recruited in the urban capital city of Yerevan than in the surrounding more rural provinces of Armavir and Lori. Women who filled out the survey were then asked if they would like to participate in a qualitative interview discussing the topics of the survey more in depth and those who agreed were then directed to complete the informed consent process for the interview.

The one-on-one interviews with women took place only in the surrounding provinces and were conducted in private rooms at the partnering NGO centers. Interviews with key informants took place at a location of the interviewee's choosing, often at their workplace, in Yerevan, Gyumri, Shirak, Lori, and Geghartunik. The survey data were collected over 2 months (June–July 2019) using the Qualtrics software. Results were analyzed using descriptive statsitical methods such as graphical displays of categorical and continous data, and are mainly presented as tables and frequencies. Subanalyses were done by conducting chi-squared tests for independence.

## Results

### Survey results

A total of 112 participants signed the informed consent form and contributed to the survey. Some participants skipped questions, and therefore question specific participant counts are reported throughout the results. A demographic breakdown of the survey participants is provided in Table 1. It is important to note that the majority of the survey participants were residing in urban communities and were highly educated.

**Table 1.** Participant demographic characteristics

Survey participants	
Age: median years (*n* = 97)	31
Highest level of education (*n* = 104), *n* (%)	
Elementary level	0 (0)
High school level	8 (7.7)
College/undergraduate level	12 (11.5)
Graduate/professional level	66 (63.5)
Post professional level (MD, DO, DDS, PhD)	15 (14.4)
Other	3 (2.9)
Current relationship status (*n* = 104), *n* (%)	
Married	48 (46.2)
Single	36 (34.6)
Widowed	9 (8.7)
Steady partner (not cohabitating)	6 (5.8)
Divorced/separated	4 (3.8)
Steady partner (cohabitating)	1 (0.9)
Ever been sexually active (*n* = 104), *n* (%)	80 (76.9)
Currently sexually active (*n* = 75), *n* (%)	50 (67)
Ideal number of children: median (*n* = 88)	3
Key informant interview participants (*n* = 15), *n* (%)	
Gender	
Male	3 (20)
Female	12 (80)
Occupation	
Obstetrician-Gynecologist	11 (73.3)
General practitioner	1 (6.7)
Sexologist	1 (6.7)
Nurse	1 (6.7)
Endocrinologist	1 (6.7)
Province of practice	
Lori	2 (13.3)
Gyumri	3 (20)
Shirak	1 (6.7)
Geghartunik	3 (20)
Yerevan	6 (40)

Overall, a total of *N* = 112 women participated in the survey, however, not every participant completed each question; therefore, question specific participant numbers are reported in the table above, and percentages are calculated with a denominator of the question specific number of participants.

**FIG. 1.**
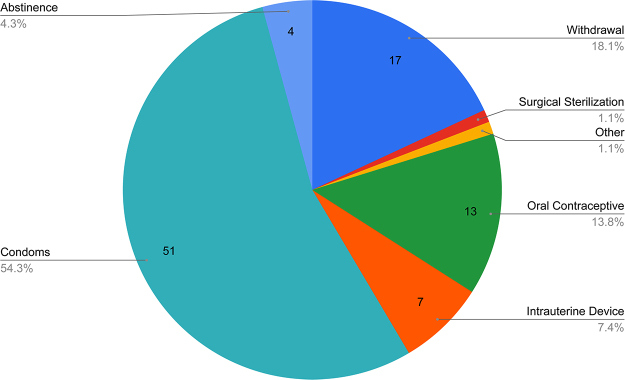
Types of contraception ever used by participants. Figure summarizes contraceptive methods that have ever been used by those 57 survey participants who indicated that they have ever used some form of contraception.

**FIG. 2.**
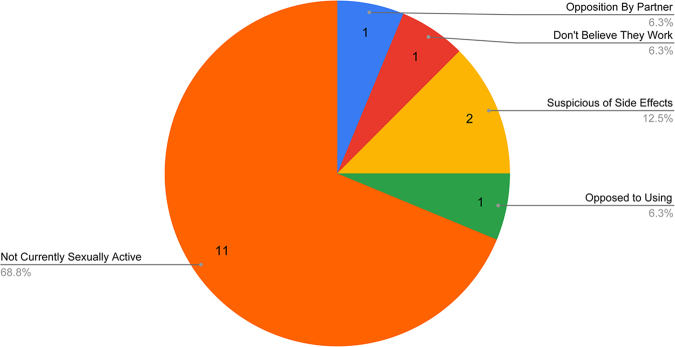
Reasons for disuse of contraception. Figure summarizes the reasons for not using contraception by those 16 survey participants who indicated they do not use any form of contraception.

Of the survey participants answering this set of questions (*n* = 78), 73% have used a form of contraception during their lifetime, and of those (*n* = 57), 66% are currently using contraception. The types of contraception ever used by participants are summarized in Figure 1 (*n* = 57) with the leading method ever used being condoms. Participants' reasons for not currently using any form of contraception are detailed in Figure 2 (*n* = 16), often citing not to be currently sexually active. For the purposes of this survey, “modern” methods consisted of oral contraceptive pills, intrauterine devices, condoms, surgical sterilization, spermicides, vaginal rings, and hormonal injects/patches/implants. “Traditional” methods were defined as withdrawal or fertility cycle planning. Participants were also provided with a free response option of “other” to be utilized as needed.

When ranking contraceptive methods known to participants (*n* = 82) according to their preferences (each awarding 5 points for the highest ranked choice and 1 point for the lowest ranked choice of 5 options), modern contraception (including condoms) received the most support (346 ranking points), followed by traditional methods (237 ranking points), abstinence (176 points), abortion (134 points), and finally, other methods (44 points). When inquiring about participants' knowledge of various contraceptive methods (*n* = 92), the majority was familiar with condoms (*n* = 87), intrauterine devices (*n* = 78), and oral contraceptive pills (*n* = 70). The least familiar contraceptive methods were hormonal injections/patches and spermicides. No significant relationship between marital status and knowledge of methods was found (*p* = 0.997).

On a scale of 0–10 (with 0 being not at all effective and 10 being 100% effective in preventing pregnancy), participants rated the effectiveness of the oral contraceptive pill to be, on average, 6.88 (*n* = 92), and the effectiveness of withdrawal to be, on average, 5.31 (*n* = 88). On a scale of 0–10 (0 being “I am strongly against the use of modern contraception,” and 10 being “I am strongly in support of the use of modern contraception”), responses reached a mean of 6.13 (*n* = 86). The leading opinion in support of modern contraception was their protection against unplanned pregnancies (*n* = 10), and the leading opposing opinion was the presumption of harmful side effects (*n* = 9).

**FIG. 3.**
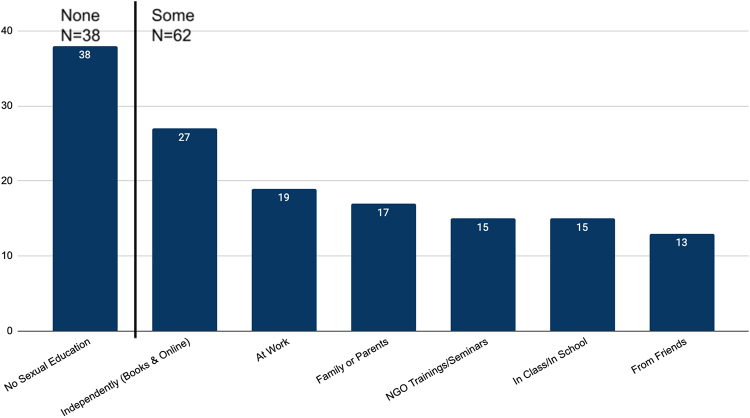
Participant sexual education. Figure summarizes the responses of those 100 survey respondents who indicated having either none or some form of sexual education. Further, the sources of sex education for the 62 participants who indicated having some form of sexual education are summarized in the figure based on frequency of selection.

Of the respondents for this question (*n* = 100), 38% reported not having received any form of sexual education, and among the remaining 62% (*n* = 62) educating themselves *via* the internet or books was most often cited (Fig. 3). The median age at first sexual education was 15.5 years.

If experiencing an unintended pregnancy, 58% of the respondents (*n* = 90) would not consider abortion as an option. However, of the same group of participants (*n* = 90), 72% believe that it is important for women to access to safe abortions. Regarding access to contraception, 94% of respondents (*n* = 95) are aware of where to get contraception, and 77% of respondents (*n* = 95) are aware of where to access a safe abortion. Marital status showed no significant relationship with access to modern contraception (*p* = 0.151), but a significant relationship between marital status and access to safe abortions (*p* = 0.007) was found when comparing women who have ever been married (married, divorced, and widowed women) to women who have never been married.

**FIG. 4.**
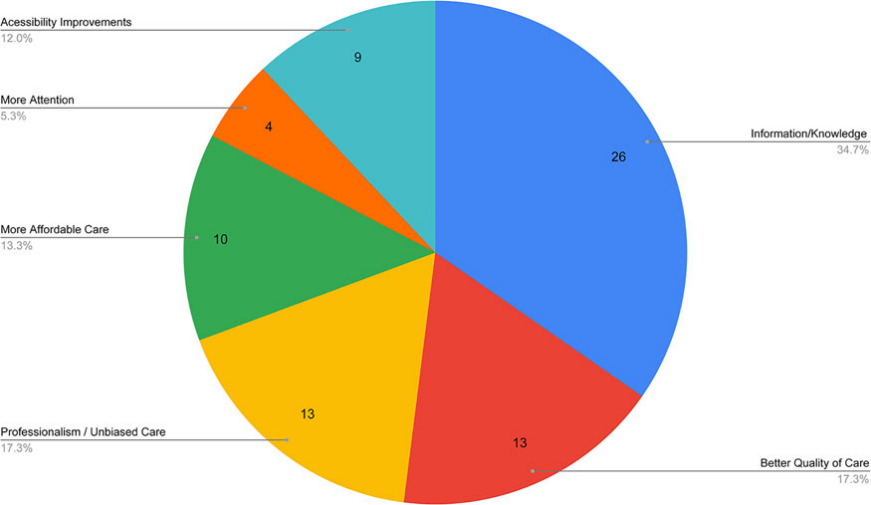
Participants' health care needs. Figure summarizes the health care needs stated by 62 survey participants.

In general, 64.63% participants (*n* = 82) stated that it would be “very easy” to obtain contraception. Figure 4 summarizes the participants' responses (*n* = 62) regarding their reproductive health needs to successfully fulfill their family planning goals. More knowledge and information was the most pressing need stated by 34.7% of women, followed by both professional unbiased care and better quality of care each stated by 17.3% of women.

### Women's interviews results

**FIG. 5.**
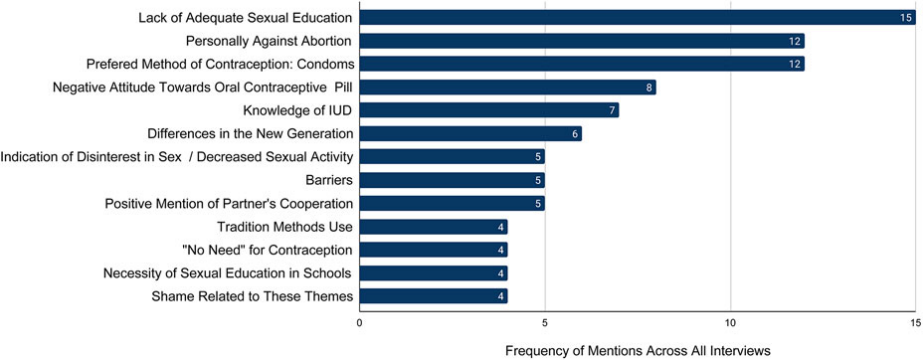
Recurring themes in women's interviews. Figure summarizes the themes that arose in the one-on-one interviews with 12 women by frequency of mentions across all interviews.

Figure 5 (*n* = 12) summarizes the major themes during the semistructured one-on-one interviews with women. The leading concern of interviewed women was the lack of adequate sexual education.

### Key informant interview results

This study also included the voices of various key informants (*n* = 15) in the field of public health from both the urban capital city of Yerevan and the surrounding more rural provinces. Their demographics are summarized in Table 1.

**FIG. 6.**
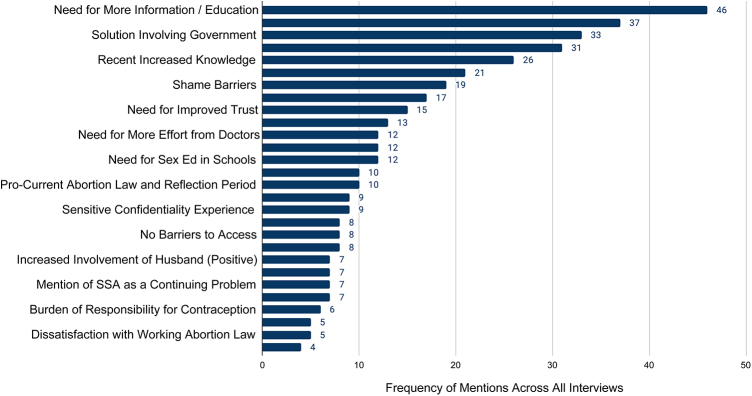
Recurring themes in key informant interviews. Figure summarizes the themes arising in the one-on-one interviews with 15 key informants by frequency of mentions across all interviews.

Figure 6 (*n* = 15) shows all major themes arising during the interviews. Discrepancies between rural provinces and the urban capital city of Yerevan in the provision of care was cited as the leading cause of concern, followed by the need to provide information and the government's responsibility in this field. Other main themes described concerns about cultural and societal issues and their role affecting reproductive health, and the acknowledgment of the recent positive changes in women's behaviors and health.

## Discussion

Although limited in scope, our results add valuable data on reproductive health, knowledge, and attitudes regarding family planning options in Armenia.

First, assessing the current use of contraception in Armenia, our results suggest an increased use of any contraceptive method in recent years (66% of study participants), while the last DHS Armenia survey in 2015 reported only 36.7% of Armenian women using some form of contraception.^6^ Our study participants primarily relied on male condoms as their main method (62%), a change compared to two previous studies stating the primary method of contraception at that time to have been withdrawal.^5,13^ While historically Armenia had a strong reliance on and preference for traditional contraceptive methods over modern ones, our participants now ranked modern contraception as their preferred choice.^5^

Our findings regarding reasons for nonuse of contraception are in line with the Westoff study from 2002: more than two-thirds of participants noted their leading reason for nonuse to be that they are not sexually active at this time, followed by opposition of use by partner (12.5%). Our survey and interview findings were also in line with the Guttmacher Institute's report of Unmet Need for Contraception in Developing Countries which found that a significant proportion (79%) of married women in Armenia cite sexual inactivity as a reason for not using contraception report that their husbands are away or staying elsewhere.^15^ Women in our study were familiar with a variety of contraceptive methods, and their marital status as a proxy for sexual experience did not seem to impact this.

Study participants rated the effectiveness of oral contraceptive pills to be fairly high (mean 6.88 of scale 0–10), and described overall positive attitudes toward oral contraception (mean 6.13). These findings suggest that women's attitudes toward contraception have improved over the last two decades, compared to women expressing predominantly negative attitudes toward contraception and believing that contraception was unreliable or unsafe.^5^

Sexual education seems to have seen much less progression over the last 20 years. While almost two-thirds of our study participants had some form of sexual education, most of them received it through independent searches online and books. Sexual education in school was often described as limited to the basics of anatomy and physiology, and lacking information on pregnancy prevention, sexually transmitted infections, consent, and healthy relationships. Women expressed a clear need for adequate sexual education, and key informants identified the lack of information on reproductive health issues to be the largest barrier Armenian women face in this field.

It is crucial to acknowledge that our sample comprised highly educated individuals and may not accurately represent the broader Armenian population. Despite this limitation, the Guttmacher Institute's report generally suggests that women with fewer years of education tend to experience higher levels of unmet contraceptive need than their more educated counterparts. Notably, Armenia stands out as one of the few countries where the levels of unmet need are at least five percentage points higher among more educated women.^15^ The observed finding in Armenia, where higher levels of education do not seem to provide the expected protection against unmet contraceptive needs as seen in other countries, suggests a potential gap in the integration of comprehensive sexual health education throughout the educational system.

This discrepancy may highlight the need for targeted efforts to ensure that education equips individuals with the necessary knowledge and resources to make informed decisions about contraception, thereby addressing unmet needs across diverse educational backgrounds.

Abortion has been legal for decades and widely available in Armenia throughout the years of the soviet era. Almost three-quarters (73%) of our study participants believed in the importance of access to safe abortions and a woman's right to choose. However, only 43% of those same participants would consider abortion an option for themselves, a clear departure from earlier times when at least 50% of women would consider an abortion in case of an unintended pregnancy.^5^ This finding suggests that the belief in access to safe abortions as a woman's right continues to be anchored in Armenian society.

Discussions with our key informants support earlier observations of a decline in abortion over the past 15 years and a gradual adoption of modern contraceptive methods in recent years, a finding Westoff et al. attribute to the postponement of marriage.^5^ While we are able to conclude a general decline in abortions based on ecological data, participant responses and key informant observations, it is important to note that in all populations, the highly stigmatized nature of abortion may play a role in underestimating the populations true willingness to utilize abortion resources.

Regarding access and needs for reproductive health services, the overwhelming majority of participants were aware of where to get contraception, and almost two-thirds of respondents considered access “very easy,” independently of marital status. As in earlier studies, financial reasons were stated as impacting access.^1–3^ In addition, more than three-quarters of survey respondents were aware of where to access a safe abortion, especially among women formerly or currently married. Information and knowledge regarding reproductive health, as well as more affordable and professional care emerged as the main unmet needs, a finding reiterated in the one-on-one interviews with women and key informants alike, who emphasized the government's responsibility for creating a centralized system for sexual education in schools or through national programs.

While our results can fill a number of gaps and provide contemporary data, this study has a number of limitations. Many questions were personal and sensitive in nature (such as abortion), and nuances may have been lost in translation of the survey from English to Armenian.

In addition, while the survey was completed by a sizable number of women (*n* = 112), the sample size for one-on-one interviews with women (*n* = 12) and key informants (*n* = 15) were both fairly small. The interviews with women were only conducted in 2 rural of 11 provinces in Armenia, while the quantitative survey was mainly completed by women in the capital city of Yerevan who often had access to the internet. Compared to the general Armenian population, the survey participants were more educated and likely had access to sexual and reproductive health care through Women's Resource Centers, our main recruitment sites.

In conclusion, the results of this study include voices of women and health care providers in the field of women's reproductive health in Armenia, and suggest that over that last two decades, knowledge regarding family planning options in Armenia has increased and preferences have shifted toward modern contraceptive methods, but the need for modernized, comprehensive and culturally sensitive sex positive education persists. An instituted program providing this education could help to destigmatize the discussion about sexual health and provide reliable information to the next generation. This recommendation was supported by interviewed health care providers and women alike. Supplementing sex education in schools, we recommend continued educational and counselling programs for women throughout their life cycle within the health care system to provide adaptive, professional, and continued support for women and couples.

While this project was inherently limited in scope, it does illustrate the need for more comprehensive reviews of the existing landscape of family planning in Armenia on a much larger scale and in each province, and the evaluations of existing programs for their effectiveness in increasing women's knowledge of their reproductive options.

Finally, anthropological research is needed on societal changes over time and contributing factors, focusing on understanding the continued impact of a conservative and traditional culture on modernizing sexual and reproductive health policies.

While these recommendations may also be relevant for neighboring post-Soviet, Transcaucasian, and Middle Eastern countries, further studies are needed to both continue documenting progress and to describe drivers of women's decision-making related to family planning in these populations.

## Ethical Approval

The study received ethics approval from the Office for Protection of Human Subjects (OPHS) at the University of California at Berkeley (Protocol ID: 2019-03-11898).

## Availability of Data and Materials

Due to the sensitive nature of the questions asked in this study, survey respondents and interview participants were assured that raw data would remain confidential and would not be shared.

## Abbreviations Used

NGO: nongovernmental organization
OPHS: Office for Protection of Human Subjects
PI: principal investigator
WRCA: Women's Resource Center Armenia
