# Supplementary material for: Knowledge and Attitudes Regarding Family Planning Options in Armenia
Source: Womens Health Rep (New Rochelle). 2024 Apr 26;5(1):376–84. doi: 10.1089/whr.2024.0005 (PMC11375319; doi:10.1089/whr.2024.0005)
Supplement: Supplementary Data S1 [file whr.2024.0005_supp_datas1.pdf]

ՀԵՏԱԶՈՏՄԱՆԸ ՄԱՍՆԱԿՑԵԼՈՒ ՀԱՄԱՁԱՅՆՈՒԹՅՈՒՆ

Ընտանիքի պլանավորման ընտրանքների վերաբերյալ մոտեցումները և գիտելիքները Հայաստանում

Ներածություն

Ես Լարա Ռոստոմեանն եմ: Ես բակալավրիատի ուսանող եմ, ով աշխատում է Բերկլիի Կալիֆոռնիայի համալսարանի հանրային առողջության դպրոցում դոկտոր Անկե Հեմերլինգի (բ.գ.թ., դոցենտ) հետ:

Մենք հրավիրում ենք Ձեզ մասնակցելու այս ուսումնասիրությանը, քանի որ մենք գնահատում ենք ձեր կարծիքը որպես հայ կին և կարևորում ենք ձեր սեռական և վերարտադրողական առողջությունը: Մենք ուզում ենք իմանալ Հայաստանում ընտանեկան պլանավորման ցանկացած տարբերակների մասին, որոնք Ձեզ հայտնի են և հետաքրքիր Ձեզ համար:

Նպատակը

Այս հետազոտության նպատակն է հասկանալ Հայաստանում ներկայիս ընտանեկան պլանավորումը՝ ի վերջո օգնելու բարելավել ժամանակակից հակաբեղմնավորիչների մատչելիությունը և հետագայում նպաստել Հայաստանում ընտանեկան պլանավորման արդյունավետ նպատակների իրականացմանը: Մասնավորապես, մեր նպատակները ներառում են ժամանակակից հակաբեղմնավորիչների նկատմամբ կանանց գիտելիքների ու մոտեցումների գնահատումը, բացահայտումը, խոչընդոտների և մարտահրավերների նկարագրումը, որոնց բախվում են կանայք հակաբեղմնավորիչներ ձեռք բերելիս և ժամանակակից հակաբեղմնավորիչների վերաբերյալ կանանց ներկայիս գիտելիքների, վերաբերմունքի պոտենցիալ փոփոխությունների վերլուծությունը 1998 թ.-ից առկա տվյալների հետ համեմատության միջոցով:

Ընթացակարգեր

Եթե դուք համաձայն եք լինել այս ուսումնասիրության մեջ, ձեզանից կպահանջվի կատարել հետևյալը.

Ձեզանից կպահանջվի լրացնել հակաբեղմնավորման և ընտանեկան պլանավորման ժամանակակից և այլ մեթոդների վերաբերյալ Ձեր նախնական գիտելիքների և վերաբերմունքի մասին հարցաթերթիկ: Այս հարցաթերթիկը նաև կպարունակի հարցեր սեռական առողջության ձեր փորձի մասին: Հարցաթերթիկը թղթային տարբերակով է և դուք կգրեք ձեր պատասխանները: Ցանկության դեպքում, հարցաթերթիկը հասանելի է նաև առցանց: Եթե դուք թարգմանությունների կամ ընթերցման և պատասխանների կարիք ունեք, ապա այդ ծառայությունները ձեզ կտրամադրվեն: Դա կտնի 15-20 րոպե:

Ուսումնասիրության վայրը

Ուսումնասիրության բոլոր ընթացակարգերը տեղի կունենան «Կանանց ռեսուրս կենտրոն» ՓԲԸ-ում, որը տեղակայված է Մարշալ Բաղրամյան 50 հասցեում, ք. Երևան, Հայաստան:

Օգուտները

Այս ուսումնասիրությանը մասնակցելու ուղղակի օգուտներ չկան, սակայն հուսով ենք, որ հետազոտության արդյունքում ձեռք բերված տեղեկատվությունը կնպաստի արդյունավետ սեռակրթության զարգացմանը և ընտանիքի պլանավորման և Հայաստանում հակաբեղմնավորման ժամանակակից մեթոդների մասին կանանց իրազեկվածության բարձրացմանը:

Ռիսկեր/անհանգստություն

- հետազոտական որոշ հարցերը կարող են ձեզ անհարմարություն պատճառել կամ հուսահատություն: Դուք կարող եք հրաժարվել պատասխանել ցանկացած հարցին, որին չեք ցանկանում պատասխանել կամ դադարեցնել հարցազրույցը ցանկացած պահի:
- Գաղտնիության խախտում. ինչպես բոլոր հետազոտությունների դեպքում, կա հավանականություն, որ գաղտնիությունը կխախտվի, սակայն մենք նախազգուշական միջոցներ ենք ձեռնարկում այս ռիսկը նվազեցնելու համար:

Գաղտնիություն

Ձեր ուսումնասիրության տվյալները կկիրառվեն հնարավորինս գաղտնի: Եթե սույն ուսումնասիրության արդյունքները հրապարակվեն կամ ներկայացվեն, անհատական անուններ և այլ անձնական տեղեկություններ չեն օգտագործվի:

Գաղտնիության ռիսկերի նվազեցման համար մենք կկատարենք հետևյալը.

- Մենք չենք միավորի ձեր ինքնության և հետազոտության տվյալները: Ձայնագրություններից անմիջապես հետո անհատական նույնացուցիչները կջնջվեն: Հարցաթերթիկների վրա չի հայտնաբերվի որևէ ճանաչելի տեղեկատվություն:
- Ձեր հետազոտական գրառումները, ներառյալ աուդիո ձայնագրությունները, կպահվեն համակարգչում՝ պաշտպանված գաղտնաբառով:
- Միայն իմ ֆակուլտետի խորհրդատուն և ես կունենանք հասանելիություն ձեր հետազոտական գրառումներին:

Մենք կպահպանենք ձեր ուսումնասիրության տվյալները հնարավորինս գաղտնի, միայն եթե դա որոշակի տեղեկատվություն չէ, որը պարտավոր ենք հաղորդել իրավական կամ էթիկական պատճառներով, ինչպես օրինակ, երեխաների, ծերերի նկատմամբ դաժան վերաբերմունքը կամ ինքներդ Ձեզ կամ այլոց վնաս պատճառելու մտադրությունը:

Ուսումնասիրության տվյալների ապագայում օգտագործումը.
Հետազոտության տվյալները կպահպանվեն իմ կամ ուրիշների կողմից իրականացվող ապագա հետազոտություններում հնարավոր օգտագործման համար: Ես կպահպանեմ այս տվյալները ուսումնասիրության ավարտից մինչեւ 1 տարի: Վերոնշյալ գործողությունները կձեռնարկվեն այս ուսումնասիրության տվյալների գաղտնիությունը պահպանելու համար:

Փոխհատուցում/վճարում
Դուք չեք փոխհատուցվի այս ուսումնասիրությանը մասնակցելու համար:

Ծախսերը
Դուք չեք վճարում ուսումնասիրության որևէ գործողության համար:

Իրավունքները
Հետազոտությանը մասնակցելը լիովին կամավոր է:
Դուք իրավունք ունեք հրաժարվել մասնակցել հարցմանը կամ հրաժարվել մասնակցությունից այս հետազոտության ցանկացած պահից, առանց տուգանքի կամ օգուտի կորստի, որի հանդեպ դուք իրավունք ունեք հակառակ դեպքում:

Հարցեր
Այս ուսումնասիրության վերաբերյալ որևէ հարց կամ մտահոգություն ունենալու դեպքում կարող եք կապնվել Լարա Ռոստոմեանի հետ armeniafamilyplanning@gmail.com:
Եթե ձեր իրավունքների վերաբերյալ որևէ հարց կամ մտահոգություն ունեք, նաև հետազոտության առարկայի վերաբերյալ, կարող եք դիմել UC Berkeley- ի Մարդկային պաշտպանության կոմիտեի գրասենյակ հետևյալ հեռախոսահամարով՝ 510-642-7461 կամ էլ. փոստի միջոցով՝ subjects@berkeley.edu:

Համաձայնություն
Եթե ցանկանում եք մասնակցել այս ուսումնասիրությանը, խնդրում ենք լրացնել այս հարցաշարը և ավարտելուն պես այն հանձնել Լարա Ռոստոմեանին կամ թողնել նշված վայրում՝ Հայաստանի Կանանց Կենտրոնում:

1. Նշե՛ք ձեր տարիքը: ☐

2. Նշե՛ք ձեր բարձրագույն կրթական մակարդակը:

- O Թերի միջնակարգ
- O Միջնակարգ
- O Միջին մասնագիտական
- O Բարձրագույն
- O Հետբուհական մասնագիտական աստիճան
- O Այլ____

3. Ինչպիսի՞ն է ձեր ներկա հարաբերությունները:

- O Ամուսնացած
- O Ամուսնալուծված
- O Այրի
- O Կայուն զուգնկեր միասին ապրելով
- O Կայուն զուգնկեր առանձին ապրելով
- O Միայնակ
- O Այլ ____

4. Հետևյալներից ո՞րն է լավագույնս նկարագրում ձեր ապրելակերպը : [Ընտրեք ձեր համար բոլոր կիրառելի պատասխանները]

- ☐ Ապրում եք միայնակ
- ☐ Ապրում եք ընկերոջ / ընկերների հետ
- ☐ Ապրում եք ձեր զուգընկերոջ հետ
- ☐ Ապրում եք ձեր ծնողների / անմիջական ընտանիքի հետ
- ☐ Ապրում եք ձեր զուգընկերոջ ծնողների հետ
- ☐ Ապրում եք ձեր երեխաների հետ
- ☐ Այլ ____

5. Դուք երբևէ ունեցե՞լ սեռական հարաբերություն: [Եթե ոչ, անցեք 13 հարցին]

- O Այո
- O Ոչ

6. Դուք ներկա պահին վարում ե՞ք ակտիվ սեռական կյանք

O Այո
O Ոչ

7. Քանի՞ հղիություն եք ունեցել: [Եթե ոչ, տեղադրել 0]

Կենդանածին__________
Վիժում_________
Աբորտ__________
Ընդհանուր_________

8. Դուք երբևէ օգտագործե՞լ եք հակաբեղմնավորման որևէ ձև:

O Այո
O Ոչ

9. Եթե այո, ապա նշվածներից որոնք: [Ընտրեք ձեր համար բոլոր կիրառելի պատասխանները]

☐ Հակաբեղմնավորիչ դեղեր
☐ Ներարգանդային պարույր
☐ Պահպանակ
☐ Արտաքին սերմնաժայթքում
☐ Վիրաբուժական ստերիլիզացում զույգերից մեկի կամ մյուսի մոտ
☐ Հարաբերություն չունենալ
☐ Այլ____

10. Ներկա պահին օգտագործե՞լ եք հակաբեղմնավորման որևէ ձև

O Այո
O Ոչ

11. Եթե այո, ապա ինչ տեսակի: [Ընտրեք ձեր համար բոլոր կիրառելի պատասխանները]

☐ Հակաբեղմնավորիչ դեղեր
☐ Ներարգանդային պարույր
☐ Պահպանակ
☐ Արտաքին սերմնաժայթքում
☐ Վիրաբուժական ստերիլիզացում զույգերից մեկի կամ մյուսի մոտ

☐ Հարաբերություն չունենալ

☐ Այլ ____

12. Եթե դուք պատասխանել " ոչ " 8 և / կամ 10 հարցերին, ապա Ինչու ոչ? [Ընտրեք ձեր համար բոլոր կիրառելի պատասխանները]

- O Ես չգիտեմ, թե որտեղից ձեռք բերեմ դրանք
- O Դրանք շատ թանկ են
- O Ես փորձում եմ հղիանալ այս պահին
- O Ես կասկածում եմ կողմնակի ազդեցություններին և առողջական խնդիրներ ունեմ
- O Ես չեմ հավատում, որ նրանք իսկապես աշխատում են
- O Ես դեմ եմ հակաբեղմնավորիչների օգտագործմանը
- O Իմ ամուսինը(զուգընկերը) դեմ է, որ ես օգտագործեմ հակաբեղմնավորիչ
- O Ես ներկա պահին վարում եմ պասիվ սեռական կյանք
- O Այլ ____

13. Հակաբեղմնավորման որ մեթոդների մասին գիտեք: [Ընտրեք ձեր համար բոլոր կիրառելի պատասխանները]

- O Հակաբեղմնավորիչ դեղեր
- O Ներարգանդային պարույր
- O Պահպանակ
- O Արտաքին սերմնաժայթքում
- O Վիրաբուժական ստերիլիզացում
- O Հարաբերություն չունենալ
- O Պտղաբերության ցիկլ պլանավորում
- O Սպերմացիդ
- O Հորմոնալ ներարկումներ
- O Հեշտոցային օղակ
- O Այլ ____________________________

14. Գիտե՞ք, թե ուր կարելի է դիմել հակաբեղմնավորիչ միջոցների համար

- O Այո
- O Ոչ

15. Գիտե՞ք, թե որտեղ պետք է գնաք անվտանգ աբորտի համար

- O Այո
- O Ոչ

15. Ձեր կարծիքով, որքանո՞վ են արդյունավետ խմելու համար նախատեսված հակաբեղմնավորիչ դեղերը հղիության կանխարգելման համար: (0 – արդյունավետ չէ 10-ը 100% արդյունավետ)

0 1 2 3 4 5 6 7 8 9 10

16. Ձեր կարծիքով, որքանո՞վ է արդյունավետ արտաքին սերմնաժայթքման մեթոդը, որպես հակաբեղմնավորման մեթոդ կանխելով հղիությունը: (0 – արդյունավետ չէ 10-ը 100% արդյունավետ է)

0 1 2 3 4 5 6 7 8 9 10

17. Դուք երբևէ ունեցել եք սեռական կրթության:

- O Այո
- O Ոչ

18. Եթե այո, ապա որտե՛ղ և ու՛մ կողմից: [Ընտրեք ձեր համար բոլոր կիրառելի պատասխանները: [Խնդրում ենք թողնել դատարկ, եթե դուք պատասխանել եք "ոչ" 17-րդ հարցին]:

- O Իմ ընկերներից
- O Իմ ընտանիքից կամ ծնողներից
- O Դասարանում / դպրոցում
- O Առցանց
- O Աշխատավայրում
- O Այլ____

19. Ո՞ր տարիքում եք առաջին անգամ ստացել սեռական կրթություն: [Խնդրում ենք թողնել դատարկ, եթե դուք պատասխանել եք "ոչ" 17-րդ հարցին] ☐

20. ինչպե՞ս եք վերաբերվում ժամանակակից հակաբեղմնավորիչներին (օրինակ, խմելու համար նախատեսված հաբեր, ներարկումներ, ներարգանդային պարույր)

0 նշելու դեպքում դուք կտրականապես դեմ եք իրենց օգտագործմանը 5 նշելու դեպքում չեզոք, 10 նշելու դեպքում ամբողջությամբ կողմ եմ :

0 1 2 3 4 5 6 7 8 9 10

21. Ինչու՞

____

22. ինչպե՞ս է ձեր ամուսինը / զուգընկերը վերաբերվում ժամանակակից հակաբեղմնավորիչներին (օրինակ, խմելու համար նախատեսված հաբեր, ներարկումներ, ներարգանդային պարույր)

0 նշելու դեպքում կտրականապես դեմ է օգտագործմանը 5 նշելու դեպքում չեզոք, 10 նշելու դեպքում ամբողջությամբ կողմ է :

0 1 2 3 4 5 6 7 8 9 10

23. Ինչու

____

24. ինչպիսի՞ն է ձեր ծնողների վերաբերմումունքը ժամանակակից հակաբեղմնավորիչների վերաբերյալ (օրինակ, խմելու համար նախատեսված հաբեր, ներարկումներ, ներարգանդային պարույր)

0 նշելու դեպքում կտրականապես դեմ է օգտագործմանը 5 նշելու դեպքում չեզոք, 10 նշելու դեպքում ամբողջությամբ կողմ է:

0 1 2 3 4 5 6 7 8 9 10

25. Ի՞նչ քանակությամբ երեխաները կլինեն իդեալական Ձեզ համար. ☐

26. Ի՞նչ է ձեզ անհրաժեշտ առողջապահական համակարգից, ընտանիքի պլանավորման նպատակներին հասնելու համար:

____

____

27.Կարծում եք, որ կանանց համար կարևո՞ր է աբորտի մատչելիությունը և հասանելիությունը:

- O Այո
- O Ոչ

28. Ինչու՞ այո և ինչու՞ ոչ

____

____

CPHS 2019-0311898

29. Եթե չնախատեսված հղիություն ունենայիք, կնախընտրեիք աբորտ անել:

- O Այո
- O Ոչ

30. Եթե Ոչ, Ապա Ինչու

____

____

31. Եթե ցանկանում եք ժամանակակից Հակաբեղմնավորիչ ձեռք բերել (խմելու համար նախատեսված հակաբեղմնավորման հաբեր, ներարգանդային պարույրներ և այլն), կարծում եք, որ դա կլինի:

- O **Շատ պարզ**
- O **Ոչ այդքան հեշտ**
- O **Շատ դժվար**
- O **Անհնար**

32. Ինչու՞

____

33. Դասավորեք ընտանիքի պլանավորման հետևյալ մեթոդները նախապատվության կարգով: [1 նախնտրելի, 5 ոչ այդքան նախընտրելի :

_____Հակաբեղմնավորման ժամանակակից մեթոդներ (պահպանակներ, խմելու համար նախատեսված հաբեր, ներարգանդային պարույրներ, հորմոնալ իմպլանտատներ և այլն)

___Ավանդական հակաբեղմնավորման մեթոդներ (արտաքին սերմնաժայթքմում, ծննդաբերության ցիկլի պլանավորում)

_____Հարաբերություն չունենալ

_____Աբորտ

_____Այլ____________________________

ՀԵՏԱԶՈՏՄԱՆԸ ՄԱՍՆԱԿՑԵԼՈՒ ՀԱՄԱՁԱՅՆՈՒԹՅՈՒՆ

Ընտանիքի պլանավորման ընտրանքների վերաբերյալ մոտեցումները և գիտելիքները Հայաստանում

Ներածություն

Ես Լարա Ռոստոմեանն եմ: Ես բակալավրիատի ուսանող եմ, ով աշխատում է Բերկլիի Կալիֆոռնիայի համալսարանի հանրային առողջության դպրոցում դոկտոր Անկե Հեմերլինգի (բ.գ.թ., դոցենտ) հետ:

Մենք հրավիրում ենք Ձեզ մասնակցելու այս ուսումնասիրությանը, քանի որ մենք գնահատում ենք ձեր կարծիքը որպես հայ կին և կարևորում ենք ձեր սեռական և վերարտադրողական առողջությունը: Մենք ուզում ենք իմանալ Հայաստանում ընտանեկան պլանավորման ցանկացած տարբերակների մասին, որոնք Ձեզ հայտնի են և հետաքրքիր Ձեզ համար:

Նպատակը

Այս հետազոտության նպատակն է հասկանալ Հայաստանում ներկայիս ընտանեկան պլանավորումը՝ ի վերջո օգնելու բարելավել ժամանակակից հակաբեղմնավորիչների մատչելիությունը և հետագայում նպաստել Հայաստանում ընտանեկան պլանավորման արդյունավետ նպատակների իրականացմանը: Մասնավորապես, մեր նպատակները ներառում են ժամանակակից հակաբեղմնավորիչների նկատմամբ կանանց գիտելիքների ու մոտեցումների գնահատումը, բացահայտումը, խոչընդոտների և մարտահրավերների նկարագրումը, որոնց բախվում են կանայք հակաբեղմնավորիչներ ձեռք բերելիս և ժամանակակից հակաբեղմնավորիչների վերաբերյալ կանանց ներկայիս գիտելիքների, վերաբերմունքի պոտենցիալ փոփոխությունների վերլուծությունը 1998 թվականից առկա տվյալների հետ համեմատության միջոցով:

Ընթացակարգեր

Եթե դուք համաձայն եք լինել այս ուսումնասիրության մեջ, ձեզանից կպահանջվի կատարել հետևյալը.

Ես ձեզ հետ հարցազրույցը կանցկացնեմ «Կանանց ռեսուրս կենտրոն» տարածքում գտնվող մի ապահով վայրում: Հարցազրույցը կներառի հարցեր հակաբեղմնավորման և ընտանեկան պլանավորման ժամանակակից և այլ մեթոդների վերաբերյալ Ձեր տեսակետների մասին: Մենք կքննարկենք նաեւ ձեր գիտելիքները սեռական առողջության մասին եւ ձեր կարծիքը աբորտների եւ ժամանակակից հակաբեղմնավորման մեթոդների վերաբերյալ: Ես նաեւ կցանկանամ տեղեկանալ Ձեր անձնական ընտանեկան պլանավորման նպատակների մասին: Հարցազրույցը կտեւի ոչ ավելի, քան 30 րոպե:

Ես ակնկալում եմ անցկացնել միայն մեկ հարցազրույց. սակայն պարզաբանումների համար անհրաժեշտ կլինի անդրադառնալ: Եթե այո, ապա ձեզ հետ կապվելու եմ էլ. փոստով/հեռախոսով՝ անհրաժեշտության դեպքում հարցնելու համար:

- Ձեր թույլտվությամբ ես կձայնագրեմ և գրառումներ կկատարեմ հարցազրույցի ընթացքում: Սա արվում է ձեր կողմից տրամադրված տեղեկատվությունը հստակ արձանագրելու նպատակով և կօգտագործվի միայն արձանագրության նպատակով: Եթե դուք գերադասում եք չձայնագրվել, ես փոխարենը գրառում կկատարեմ: Եթե դուք համաձայն եք ձայնագրությանը, սակայն հարցազրույցի ժամանակ անհարմար եք զգում, ես կարող եմ անջատել ձայնագրիչը ձեր խնդրանքով: Եթե ցանկանում եք բաց թողնել որևէ հարց, ապա կարող եք խնդրեք դա անել, եթե ցանկանում եք դադարեցնել ձեր մասնակցությունը ցանկացած պահի, դուք կարող եք դադարեցնել հարցազրույցը ցանկացած ժամանակ:

Ուսումնասիրության վայրը

Ուսումնասիրության բոլոր ընթացակարգերը տեղի կունենան «Կանանց ռեսուրս կենտրոն», որը տեղակայված է Մարշալ Բաղրամյան 50 հասցեում, ք. Երևան, Հայաստան:

Օգուտները

Այս ուսումնասիրությանը մասնակցելու ուղղակի օգուտներ չկան, սակայն հուսով ենք, որ հետազոտության արդյունքում ձեռք բերված տեղեկատվությունը կնպաստի արդյունավետ սեռակրթության զարգացմանը և ընտանիքի պլանավորման և Հայաստանում հակաբեղմնավորման ժամանակակից մեթոդների մասին կանանց իրազեկվածության բարձրացմանը:

Ռիսկեր/անհանգստություն

- հետազոտական որոշ հարցերը կարող են ձեզ անհարմարություն պատճառել կամ հուսահատություն: Դուք կարող եք հրաժարվել պատասխանել ցանկացած հարցին, որին չեք ցանկանում պատասխանել կամ դադարեցնել հարցազրույցը ցանկացած պահի:
- Գաղտնիության խախտում. ինչպես բոլոր հետազոտությունների դեպքում, կա հավանականություն, որ գաղտնիությունը կխախտվի, սակայն մենք նախազգուշական միջոցներ ենք ձեռնարկում այս ռիսկը նվազեցնելու համար:

Գաղտնիություն

Ձեր ուսումնասիրության տվյալները կկիրառվեն հնարավորինս գաղտնի: Եթե սույն ուսումնասիրության արդյունքները հրապարակվեն կամ ներկայացվեն, անհատական անուններ և այլ անձնական տեղեկություններ չեն օգտագործվի:

Գաղտնիության ռիսկերի նվազեցման համար մենք կկատարենք հետևյալը.

- Մենք չենք միավորի ձեր ինքնության և հետազոտության տվյալները: Ձայնագրություններից անմիջապես հետո անհատական նույնացուցիչները կջնջվեն: Հարցաթերթիկների վրա չի հայտնաբերվի որևէ ճանաչելի տեղեկատվություն:
- Ձեր հետազոտական գրառումները՝ ներառյալ աուդիո ձայնագրությունները, կպահվեն համակարգչում՝ պաշտպանված գաղտնաբառով:
- Միայն իմ ֆակուլտետի խորհրդատուն և ես կունենանք հասանելիություն ձեր հետազոտական գրառումներին:

Մենք կպահպանենք ձեր ուսումնասիրության տվյալները հնարավորինս գաղտնի, միայն եթե դա որոշակի տեղեկատվություն չէ, որը պարտավոր ենք հաղորդել իրավական կամ էթիկական պատճառներով, ինչպես օրինակ, երեխաների, ծերերի նկատմամբ դաժան վերաբերմունքը կամ ինքներդ Ձեզ կամ այլոց վնաս պատճառելու մտադրությունը:

Ուսումնասիրության տվյալների ապագա օգտագործումը.

Աուդիո ձայնագրությունները կարձանագրվեն, իսկ ժապավենները կջնջվեն ուսումնասիրության ավարտին:

Հետազոտության տվյալները կպահպանվեն իմ կամ ուրիշների կողմից իրականացվող ապագա հետազոտություններում հնարավոր օգտագործման համար: Ես կպահպանեմ այս տվյալները ուսումնասիրության ավարտից մինչև 1 տարի: Վերոնշյալ գործողությունները կձեռնարկվեն այս ուսումնասիրության տվյալների գաղտնիությունը պահպանելու համար:

Փոխհատուցում/վճարում

Դուք չեք փոխհատուցվի այս ուսումնասիրությանը մասնակցելու համար:

Ծախսերը

Դուք չեք վճարում ուսումնասիրության որևէ գործողության համար:

Իրավունքները

Հետազոտությանը մասնակցելը լիովին կամավոր է:

Դուք իրավունք ունեք հրաժարվել մասնակցել հարցմանը կամ հանել այս կամ այն կետը:

Հարցեր

Այս ուսումնասիրության վերաբերյալ որևէ հարց կամ մտահոգություն ունենալու դեպքում կարող եք կապնվել Լարա Ռոստոմեանի հետ armeniafamilyplanning@gmail.com:

Եթե ձեր իրավունքների վերաբերյալ որևէ հարց կամ մտահոգություն ունեք, նաև հետազոտության առարկայի վերաբերյալ, կարող եք դիմել UC Berkeley- ի Մարդկային պաշտպանության կոմիտեի գրասենյակ հետևյալ հեռախոսահամարով ՝ 510-642-7461 կամ էլ. փոստի միջոցով՝ subjects@berkeley.edu:

Համաձայնություն

Դուք ստացել եք այս համաձայնության ձևի պատճենը, որպեսզի պահպանեք այն:

Եթե ցանկանում եք մասնակցել այս ուսումնասիրությանը, խնդրում ենք տեղեկացնել ինձ այդ մասին, և մենք կարող ենք շարունակել հարցազրույցը:

Հարցազրույցի թեմաներ 1:1

1. Ժողովրդագրական: Տարիք, ամուսնական / հարաբերությունների կարգավիճակ, ապրելակերպ, երեխաների թվաքանակ, զբաղվածություն:

2. Հակաբեղմնավորիչների կիրառում: Ինչպիսի՞ն է եղել ձեր նախկին փորձը հակաբեղմնավորիչների ոլորտում: Եղել է բարենպաստ, թե՞ անբարենպաստ: Ինչու՞:

3. Եթե կիրառելի է: Պատճառները ժամանակակից հակաբեղմնավորիչ միջոցներից խուսափելու:

4. Առաջին անգամ ե՞րբ է հանդիպել սեռական դաստիարակության մասին նյութեր (եթե այդպիսիք հանդիպել են):

- Որտեղ ե՞ք առաջին անգամ լսել հակաբեղմնաորիչների մասին,ո՞վ է խոսել ձեր հետ այդ թեմայով և ինչ համատեքստում:

- Ո՞ր տարիքում եք ստացել սեռական կրթություն: Ի՞նչ է այն իր մեջ ներառում :

- Արդյո՞ք այն ներառում է ուղիներ, կանխարգելելու՝ սեռական ճանապարհով փոխանցվող վարակները և հղիությունը:

- Արդյո՞ք այն ներառում է առողջ հարաբերությունների և համաձայնության ասպեկտները:

5. Քննարկել գործընկերների, ծնողների և սեփական տեսակետները ժամանակակից հակաբեղմնավորիչ միջոցները աբորտի համեմատ:

6. Ո՞րն է ձեր իդեալական ընտանիքի պլանավորման տեսլականը, ի՞նչ է նրանց պակասում դրան հասնելու համար:

7. Եթե կիրառելի է: Առողջապահական ծառայություններ մատուցողների կամ կլինիկայի աշխատակիցների հետ ուղեցույցների որոնման փորձերը հակաբեղմնավորման, աբորտի կամ ընտանեկան պլանավորման վերաբերյալ:

8. Քննարկել, թե կանայք ինչպե՞ս են սահմանում ժամանակակից հակաբեղմնավորիչ և ավանդական հակաբեղմնավորիչ միջոցները և իրենց փորձը այդ երկու հակաբեղմնավորման մեթոդների, նրանց ընկալումները դրանց արդյունավետության վերաբերյալ:

9. Քննարկել ներկա և կիրառելի եղանակով ընտանեկան պլանավորման ռեսուրսների մատչելիությունը և հասանելիությունը:

10. Քննարկել ժամանակակից հակաբեղմնավորման տարբեր մեթոդներ, եթե կանայք ծանոթ են որևէ մեկին կամ բոլորին:

ՀԵՏԱԶՈՏՄԱՆԸ ՄԱՍՆԱԿՑԵԼՈՒ ՀԱՄԱՁԱՅՆՈՒԹՅՈՒՆ

Ընտանեկան պլանավորման ընտրանքների վերաբերյալ գիտելիքները և վերաբերմունքը Հայաստանում

Ներածություն

Ես Լարա Ռոստոմյանն եմ: Ես բակալավրիատի ուսանող եմ, ով աշխատում է Բերկլիի Կալիֆոռնիայի համալսարանի հանրային առողջության դպրոցում դոկտոր Անկե Հեմերլինգի (բ.գ.թ., դոցենտ) հետ:

Մենք հրավիրում ենք Ձեզ մասնակցելու այս ուսումնասիրությանը, քանի որ մենք գնահատում ենք ձեր կարծիքը որպես կլինիկական ծառայությունների և կրթության մատակարար՝ կապված սեռական և վերարտադրողական առողջության հետ: Մենք ուզում ենք իմանալ Հայաստանում ընտանեկան պլանավորման ցանկացած տարբերակների մասին, որոնք Ձեզ հայտնի են և հետաքրքիր Ձեզ համար:

Նպատակը

Այս հետազոտության նպատակն է հասկանալ Հայաստանում ներկա ընտանեկան պլանավորումը՝ ի վերջո օգնելու բարելավել ժամանակակից հակաբեղմնավորիչ միջոցների հասանելիությունը և հետագայում նպաստել Հայաստանում ընտանեկան պլանավորման արդյունավետ նպատակների իրականացմանը:

Մասնավորապես, մեր նպատակները ներառում են կանանց գիտելիքներն ու վերաբերմունքը ժամանակակից հակաբեղմնավորիչների նկատմամբ, առկա խոչընդոտների և խղիրների բացահայտումը և նկարագրությունը, որոնց բախվում են կանայք հակաբեղմնավորիչների մատչելիության հարցում և ժամանակակից հակաբեղմնավորիչների վերաբերյալ կանանց ներկայիս գիտելիքների, վերաբերմունքի պոտենցիալ փոփոխությունների վերլուծությունը 1998 թ.-ից առկա տվյալների հետ համեմատության միջոցով:

Բացի 18-40 տարեկան հայ կանանց հարցազրույցից, մեզ համար հատկապես արժեքավոր է ձեր տեսակետը, որպես սեռական և վերարտադրողական ծառայություններ կամ կրթություն մատուցող:

Ընթացակարգեր

Եթե դուք համաձայն եք լինել այս ուսումնասիրության մեջ, ձեզանից կպահանջվի կատարել հետևյալը.

• Ես ձեզ հետ զրույցը կանցկացնեմ նախապես որոշված ժամին և ձեզ համար հարմար վայրում: Հարցազրույցը կներառի հարցեր պացիենտների գաղտնիության վերաբերյալ Ձեր տեսակետների մասին, ինչպես նաև ընտանիքի պլանավորման տարբերակները՝ ներառյալ հղիության արհեստական ընդհատումները և ժամանակակից հակաբեղմնավորման մեթոդները: Մենք կքննարկենք նաև Հայաստանում կանանց մուտքի արգելքները, որոնք առնչվում են նրանց սեռական առողջության հետ: Ես նաև կիմանամ այդ ոլորտում Ձեր պատմությունը և ապագայի փոփոխության և առաջընթացի վերաբերյալ ձեր կարծիքը: Այն կտևի ոչ ավելի, քան 45 - 60 րոպե:

- Ես ակնկալում եմ անցկացնել միայն մեկ հարցազրույց. սակայն պարզաբանումների համար անհրաժեշտ կլինի անդրադառնալ: Եթե այո, ապա ձեզ հետ կապվելու եմ էլ. փոստով/հեռախոսով՝ անհրաժեշտության դեպքում հարցնելու համար:

- Ձեր թույլտվությամբ ես կձայնագրեմ և գրառումներ կկատարեմ հարցազրույցի ընթացքում: Սա արվում է ձեր կողմից տրամադրված տեղեկատվությունը հստակ արձանագրելու նպատակով և կօգտագործվի միայն արձանագրության նպատակով: Եթե դուք գերադասում եք չձայնագրվել, ես փոխարենը գրառումներ կկատարեմ: Եթե դուք համաձայն եք ձայնագրությանը, սակայն հարցազրույցի ժամանակ անհարմար եք զգում, ես կարող եմ անջատել ձայնագրիչը ձեր խնդրանքով: Եթե ցանկանում եք բաց թողնել որևէ հարց, ապա կարող եք խնդրել դա անել: Եթե ցանկանում եք դադարեցնել ձեր

Հիմնական տեղեկատվական հարցազրույցի ուղեցույց

1. Որքա՞ն ժամանակ եք աշխատում այս ոլորտում: Ինչով եք զբաղվում:

2. Ի՞նչ փոփոխություններ եք նկատել վերջին 20 տարիների ընթացքում կանանց սեռական առողջության ոլորտում:Արդյոք այդ փոփոխությունները հիմնականում դրական են կամ բացասական:

3. Ինչպիսի՞ն էր ձեր ընդհանուր փորձը ընտանիքի պլանավորման հարցում հետաքրքրված կանանց հետ: Ինչ եք նրանց խորհուրդ տվել:

4. Ի՞նչ եք կարծում, որն է կանանց ամենամեծ խնդիրը, կապված իրենց սեռական առողջության հետ:

5. Ի՞նչն եք համարում խոչընդոտ կանանց համար, երբ ցանկանում են օգտվել ընտանեկան պլանավորման ռեսուրսներից և ժամանակակից հակաբեղմնավորիչներից:

6. Ինչպիսի՞ն է ձեր փորձը և ինչ կարծիք ունեք ընտանիքի պլանավորման խորհրդատվության համար դիմող կանանց գաղտնիության վերաբերյալ:

7. Ինչպե՞ս եք գնահատում Հայաստանում գործող աբորտների մասին օրենքները, արդյո՞ք դրանք ազդում են ընտանեկան պլանավորման վրա:

8. Դուք կարծում եք, որ աբորտը Հայաստանում ընտանիքի պլանավորման գործում կարևոր մա՞ս է կազմում:

9. Ի՛նչ եք կարծում, ինչու՞ է աբորտների մակարդակն այդքան բարձր (իսկ հակաբեղմնավորիչ միջոցների օգտագործումն այդքան ցածր) Հայաստանում կանանց շրջանում, այլ երկրների համեմատ:

10. Ի՛նչ եք կարծում, ինչպե՞ս կարող ենք ընդլայնել հակաբեղմնավորիչների օգտագործումը և նվազեցնել աբորտների անհրաժեշտությունը, որպես ընտանիքի պլանավորման մեթոդ:

11. Որտե՞ղ եք տեսնում ամենամեծ հնարավորությունները, ապագայում բարելավում մտցնելու այս ոլորտում:

12. Այլ բան կա՞, որ կցանկանայիք կիսվել այս թեմաների շուրջ:
