# Supplementary material for: Knowledge and Attitudes Regarding Family Planning Options in Armenia
Source: Womens Health Rep (New Rochelle). 2024 Apr 26;5(1):376–84. doi: 10.1089/whr.2024.0005 (PMC11375319; doi:10.1089/whr.2024.0005)
Supplement: Supplementary Data S2 [file whr.2024.0005_supp_datas2.pdf]

UNIVERSITY OF CALIFORNIA AT BERKELEY

BERKELEY • DAVIS • IRVINE • LOS ANGELES • MERCED • RIVERSIDE • SAN DIEGO | SAN FRANCISCO • SANTA BARBARA • SANTA CRUZ

CONSENT TO PARTICIPATE IN RESEARCH
Knowledge and Attitudes Regarding Family Planning Options in Armenia

Introduction

My name is Lara Rostomian. I am a undergraduate student working with Dr. Anke Hemmerling (MD, PhD, MPH) in the School Of Public Health at the University of California, Berkeley.

We are inviting you to participate in this study because we value your opinion as an Armenian woman and believe your sexual and reproductive health is important. We want to learn more about any family planning options in Armenia you are aware of and interested in.

Purpose

The purpose of this research study is to understand present-day family planning in Armenia to ultimately help improve access to modern contraception and further help progress effective family planning goals in Armenia. Specifically, our goals include to assess women's knowledge and attitudes toward modern contraception today, identify and describe the barriers and challenges women face in accessing contraception, and analyze potential change by comparing current women's knowledge and attitudes towards modern contraception to data available from 1998.

Procedures

If you agree to be in this study, you will be asked to do the following:

- You will be asked to complete a questionnaire about your previous knowledge and attitudes regarding modern contraception and other family planning methods. This questionnaire will also inquire about your sexual health practices. The questionnaire is on paper and you will write in your answers. If you would prefer, the questionnaire is also available online. If you are in need of translations or assistance in reading and responding, these services will be provided for you. This should take at most 15-20 minutes.

Study Location

All study procedures will take place at Women's Resource Center Armenia located at 50 Marshal Baghramyan Ave, Yerevan, Armenia.

Benefits

There are no direct benefits to participating in this study, however, we hope that the information gained from the study will help lead to the development of effective sexual education and outreach to inform women on various family planning methods and modern contraceptive methods in Armenia.

Risks/Discomforts

- Some of the research questions may make you uncomfortable or upset. You are free to decline to answer any questions you don't wish to or to stop the survey at any time.
- Breach of confidentiality: As with all research, there is a chance that confidentiality could be compromised; however, we are taking precautions to minimize this risk.

Confidentiality

Your study data will be handled as confidentially as possible. If results of this study are published or presented, individual names and other personally identifiable information will not be used.

To minimize the risks to confidentiality, we will do the following:

- The data will be collected anonymously. We will not maintain a link between your identity and the research data.
- Your research records (completed questionnaires) will be stored in a secure storage unit and when transcribed, on a password-protected computer.
- Only my faculty advisor and I will have access to your study records.

We will keep your study data as confidential as possible, unless it is certain information that we must report for legal or ethical reasons, such as child abuse, elder abuse, or intent to hurt yourself or others.

Future Use of Study Data

The research data will be maintained for possible use in future research by myself or others. I will retain this data for up to 1 years after the study is over. The same measures described above will be taken to protect the confidentiality of this study data.

Compensation/Payment

You will not be compensated for your participation in this study.

Costs

You will not be charged for any of the study activities.

Rights

Participation in research is completely voluntary.

You have the right to decline to participate or to withdraw at any point in this study without penalty or loss of benefits to which you are otherwise entitled.

Questions

If you have any questions or concerns about this study, you may contact Lara Rostomian at armeniafamilyplanning@gmail.com.

If you have any questions or concerns about your rights and treatment as a research subject, you may contact the office of UC Berkeley's Committee for the Protection of Human Subjects, at 510-642-7461 or subjects@berkeley.edu.

Consent

If you consent to participate in this research please complete this survey and then give it to Lara Rostomian or drop it off at the indicated location within Women's Resource Center, Armenia when done.

1. **What is your age?** ☐

2. **Which of the following best describes your level of education?**

◯ Elementary Level

◯ High School Level

◯ College / Undergraduate Level

◯ Graduate/ Professional Level

◯ Post-Professional Degree [PhD, MD, DO, DDS]

◯ Other ____

3. **What is your current relationship status?**

◯ Married

◯ Widowed

◯ Divorced/Separated

◯ Steady partner, cohabitating

◯ Steady partner, not cohabitating

◯ Single

◯ Other ____

4. **Which of the following best describes your living situation? [Select all that apply]**

☐ Living on your own
☐ Living with roommates / friends
☐ Living with your partner
☐ Living with your parents / immediate family
☐ Living with your partner's parents
☐ Living with your children
☐ Other ______________________________

5. **Have you ever been sexually active?** ***[If no, you may skip to Question 13]***

◯ Yes

◯ No

6. **Are you currently sexually active?**

○ Yes

○ No

7. **How many pregnancies have you had? [If none, please put 0]**

- Live Births :___________
- Miscarriages :__________
- Abortions :____________
- Total :________________

8. **Have you ever used any forms of contraception?**

○ Yes

○ No

9. **If so, what type? [Select all that apply]**

- ☐ Oral Contraceptive
- ☐ Intrauterine Device (IUD)
- ☐ Condoms
- ☐ Withdrawal
- ☐ Surgical sterilization of one or both partners involved.
- ☐ Abstinence
- ☐ Other ___________________________________

10. **Are you currently using any form of contraception?**

○ Yes

○ No

11. **If so, what type? [Select all that apply]**

- ☐ Oral Contraceptive
- ☐ Intrauterine Device (IUD)
- ☐ Condoms
- ☐ Withdrawal
- ☐ Surgical sterilization of one or both partners involved.
- ☐ Abstinence
- ☐ Other ___________________________________

12. If you answered no to questions 8 and/or 10, why not? [Select all that apply]

- ☐ I don’t know where to go to get it.
- ☐ It's too expensive.
- ☐ I am trying to conceive at this time.
- ☐ I am suspicious of the side effects and have health concerns.
- ☐ I don't believe they actually work.
- ☐ I am opposed to using contraception.
- ☐ My partner is opposed to my using contraception.
- ☐ I am not sexually active at this time
- ☐ Other ____

13. What methods of contraception do you know about? [Select all that apply]

- ☐ Oral Contraceptive
- ☐ Intrauterine Device (IUD)
- ☐ Condoms
- ☐ Withdrawal
- ☐ Surgical Sterilization
- ☐ Abstinence
- ☐ Fertility Cycle Planning
- ☐ Spermicides
- ☐ Hormonal Injections / Patches / Implants
- ☐ Vaginal Ring
- ☐ Other ____

14. Are you aware of locations where you can go to get contraception if you were interested in doing so?

○ Yes

○ No

15. Are you aware of locations where you can go to get a safe abortion if you were interested in doing so?

○ Yes

○ No

15. In your opinion, how effective are oral contraceptive pills in preventing pregnancy? *0 being not at all effective and 10 being 100% effective*

0 1 2 3 4 5 6 7 8 9 10

16. In your opinion, how effective is withdrawal as a contraceptive method to preventing pregnancy? *0 being not at all effective and 10 being 100% effective*

0 1 2 3 4 5 6 7 8 9 10

17. Have you ever had any form of sexual education?

○ Yes

○ No

18. If so - which describes the format of your sexual education? [Select all that apply. Please leave blank if you responded "No" to question 17]

☐ From my friends

☐ From my family or parents

☐ In class/ In school

☐ Online

☐ At work

☐ Other ____

19. At what age did you first receive sexual education? [Please leave blank if you responded "No" to question 17] ☐

20. What are your views on modern contraception (such as oral pills, injectables, and IUDs)?

0 being "I am strongly against the use of them" 5 being Neutral or No Opinion and 10 being "I am strongly in support of the use of them"

0 1 2 3 4 5 6 7 8 9 10

21. Why?

____

22. What are your partner's views on modern contraception (such as oral pills, injectables, and IUDs)?
0 being "They are strongly against the use of them" 5 being Neutral or No Opinion and 10 being "They are strongly in support of the use of them"

0 1 2 3 4 5 6 7 8 9 10

23. Why?

____

24. What are your parent's views on modern contraception (such as oral pills, injectables, and IUDs)?
0 being "They are strongly against the use of them" 5 being Neutral or No Opinion and 10 being "They are strongly in support of the use of them"

0 1 2 3 4 5 6 7 8 9 10

25. What would be the ideal number of children for you? ☐

26. What do you feel you need from your healthcare system in order to successfully reach your family planning goals?

____

27. Do you believe it is important for women to have access to abortion?

◯ Yes

◯ No

28. Why or Why Not?

____

29. If you experienced an unplanned pregnancy, would you consider abortion as an option?

◯ Yes

◯ No

30. If No, Why Not?

____

31. If you wanted to obtain modern contraception (oral contraceptive pills, IUD, etc.) do you believe this would be:

○ Very Easy

○ Possible, But Not Easy

○ Very Difficult

○ Impossible

32. Why?

____

33. Rank the following family planning methods in order of your preference:

______ Modern Contraceptive Use (Condoms, Oral Pills, IUD, Hormonal Implants, Patches Etc.)

______ Traditional Contraceptive Methods (Withdrawal, Fertility Cycle Planning)

______ Abstinence

______ Abortion

______ Other ________________

CONSENT TO PARTICIPATE IN RESEARCH
Knowledge and Attitudes Regarding Family Planning Options in Armenia

Introduction

My name is Lara Rostomian. I am a undergraduate student working with Dr. Anke Hemmerling (MD, PhD, MPH) in the School Of Public Health at the University of California, Berkeley.

We are inviting you to participate in this study because we value your opinion as an Armenian woman and believe your sexual and reproductive health is important. We want to learn more about any family planning options in Armenia you are aware of and interested in.

Purpose

The purpose of this research study is to understand present-day family planning in Armenia to ultimately help improve access to modern contraception and further help progress effective family planning goals in Armenia. Specifically, our goals include to assess women's knowledge and attitudes toward modern contraception today, identify and describe the barriers and challenges women face in accessing contraception, and analyze potential change by comparing current women's knowledge and attitudes towards modern contraception to data available from 1998.

Procedures

If you agree to be in this study, you will be asked to do the following:

- I will conduct an interview with you at a secure location within the Women's Resource Center. The interview will involve questions about your views on family planning and contraceptive methods. We will discuss your knowledge of sexual health and your opinions on abortions and modern contraceptive methods. If you are willing to share, I would also like to inquire about your personal family planning goals. It should last no longer than 30 minutes.

  I expect to conduct only one interview; however, follow-ups may be needed for clarification. If so, I will contact you by mail/phone to request this if needed.

- With your permission, I will make an audio recording and take notes during the interview. This is to accurately record information you provide, and will be used for transcription purposes only. If you choose not to be recorded, I will take notes instead. If you agree to being recorded but feel uncomfortable at any time during the interview, I can turn off the recorder at your request. If you wish to skip any given question you may ask to do so, if you wish to discontinue your participation at any time, you can stop the interview at any time.

Study Location

All study procedures will take place at Women's Resource Center Armenia located at 50 Marshal Baghramyan Ave, Yerevan, Armenia.

Benefits

There are no direct benefits of participating in this study, however, we hope that the information gained from the study will help lead to the development of effective sexual education and outreach to inform women on various family planning methods and modern contraceptive methods in Armenia.

Risks/Discomforts

- Some of the research questions may make you uncomfortable or upset. You are free to decline to answer any questions you don't wish to or to stop the interview at any time.
- Breach of confidentiality: As with all research, there is a chance that confidentiality could be compromised; however, we are taking precautions to minimize this risk.

Confidentiality

Your study data will be handled as confidentially as possible. If results of this study are published or presented, individual names and other personally identifiable information will not be used.

To minimize the risks to confidentiality, we will do the following:

- We will not maintain a link between your identity and the research data. Personal identifiers will be removed immediately after audio recordings have been transcribed. No identifiable information will be recorded on the questionnaires.
- Your research records, including audio recordings, will be stored on a password-protected computer.
- Only my faculty advisor and I will have access to your study records.

1:1 Interview Topics

1. **Demographic**: Current age, marital/relationship status, living situation, number of children, occupation.

2. Contraceptive Use: What has been your past experiences with contraception? Have they been favorable / unfavorable? Why?

3. If Applicable: Reasons for avoiding use of modern contraception

4. Experience with (if any) their first encounter of **sexual education** material.
    - Where did they first hear about contraception, who told them, and in what context?
    - At what age did they receive sexual education? What did it cover?
    - Did it cover ways to prevent STIs and Pregnancy?
    - Did it cover aspects of healthy relationships and consent?

5. Discuss partner's views, parents views, and own views on modern contraception compared to abortion.

6. What is your ideal family planning vision, what is it you are lacking to reach it?

7. If applicable: Experience with healthcare providers or clinic staff regarding your attempts to seek out contraception, abortion or family planning guidance.

8. Discuss what women define as modern contraception and traditional contraception, and their experiences with those two contraceptive methods, and their perceptions on their effectiveness?

9. Discuss access and access barriers to family planning resources if present and applicable.

10. Discuss various methods of modern contraception and if women have familiarity with any / all of them.

CONSENT TO PARTICIPATE IN RESEARCH
Knowledge and Attitudes Regarding Family Planning Options in Armenia

Introduction

My name is Lara Rostomian. I am a undergraduate student working with Dr. Anke Hemmerling (MD, PhD, MPH) in the School Of Public Health at the University of California, Berkeley.

We are inviting you to participate in this study because we value your opinion as a provider of education or clinical services related to sexual and reproductive health in Armenia.

Purpose

The purpose of this research study is to understand present-day family planning in Armenia to ultimately help improve access to modern contraception and further help progress effective family planning goals in Armenia. Specifically, our goals include to assess women's knowledge and attitudes toward modern contraception today, identify and describe the barriers and challenges women face in accessing contraception, and analyze potential change by comparing current women's knowledge and attitudes towards modern contraception to data available from 1998. In addition to interviewing Armenian women age 18-40, your view as a provider of sexual and reproductive services or education is especially valuable to us.

Procedures

If you agree to be in this study, you will be asked to do the following:

- I will conduct an interview with you at a predetermined time and secure location that is convenient for you. The interview will involve questions about your views on patient confidentiality, and family planning options including abortions and modern contraceptive methods. We will also discuss access barriers women in Armenia face regarding their sexual health. I also will inquire about your history in this field and your opinions on future change and progress. It should last no longer than 45 - 60 minutes.

I expect to conduct only one interview; however, follow-ups may be needed for clarification. If so, I will contact you by mail/phone to request this if needed.

- With your permission, I will make an audio recording and take notes during the interview. This is to accurately record information you provide, and will be used for transcription purposes only. If you choose not to be recorded, I will take notes instead. If you agree to being recorded but feel uncomfortable at any time during the interview, I can turn off the recorder at your request. If you feel uncomfortable with any given question, you may ask to skip that question. If you don't wish to continue, you can stop the interview at any time.

Benefits

There are no direct benefits of participating in this study, however, we hope that the information gained from the study will help lead to the development of effective sexual education and outreach to inform women on various family planning methods and modern contraceptive methods in Armenia.

Risks/Discomforts

- Some of the research questions may make you uncomfortable or upset. You are free to decline to answer any questions you don't wish to or to stop the interview at any time.
- Breach of confidentiality: As with all research, there is a chance that confidentiality could be compromised; however, we are taking precautions to minimize this risk.

Confidentiality

Your study data will be handled as confidentially as possible. If results of this study are published or presented, individual names and other personally identifiable information will not be used.

To minimize the risks to confidentiality, we will do the following:

- We will not maintain a link between your identity and the research data.Personal identifiers will be removed immediately after audio recordings have been transcribed. No identifiable information will be recorded.
- Your research records, including audio recordings, will be stored on a password-protected computer.
- Only my faculty advisor and I will have access to your study records.

We will keep your study data as confidential as possible, unless it is certain information that we must report for legal or ethical reasons, such as child abuse, elder abuse, or intent to hurt yourself or others.

Key Informant Interview Guide Final:

1. How long have you worked in the field? What is your occupation?
2. What changes have you observed over the past 20 years in field of women's sexual health? Have these changes been predominately positive or negative?
3. What have been your common experiences with women interested in family planning, how have you advised them?
4. What is the biggest issue you think women face regarding their sexual health?
5. What have you observed as common barriers for women when wanting to access family planning resources and modern contraception?
6. What has been your experience with and what are your views regarding confidentiality for women seeking family planning advice?
7. How do you think the current abortion laws in Armenia impact family planning choices in Armenia?
8. Do you think abortion is an important part of the family planning portfolio in Armenia?
9. Why do you think abortion rates are so high (and contraceptive use is so low) amongst women in Armenia compared to other countries?
10. What are ways you think we could increase the use of contraception and decrease the need for abortions as a method of family planning?
11. Where do you see the most opportunity in the future for improvement in this field?
12. Is there anything else you would like to share regarding these topics?
